# Variants Disrupting CD40L Transmembrane Domain and Atypical X-Linked Hyper-IgM Syndrome: A Case Report With Leishmaniasis and Review of the Literature

**DOI:** 10.3389/fimmu.2022.840767

**Published:** 2022-04-28

**Authors:** Boaz Palterer, Lorenzo Salvati, Manuela Capone, Valentina Mecheri, Laura Maggi, Alessio Mazzoni, Lorenzo Cosmi, Nila Volpi, Lucia Tiberi, Aldesia Provenzano, Sabrina Giglio, Paola Parronchi, Giandomenico Maggiore, Oreste Gallo, Alessandro Bartoloni, Francesco Annunziato, Lorenzo Zammarchi, Francesco Liotta

**Affiliations:** ^1^ Department of Experimental and Clinical Medicine, University of Florence, Florence, Italy; ^2^ Flow Cytometric Diagnostic Centre and Immunotherapy, Careggi University Hospital, Florence, Italy; ^3^ Immunology and Cell Therapies Unit, Careggi University Hospital, Florence, Italy; ^4^ Unit of Neurology and Neurophysiology, Department of Medical, Surgical and Neurological Sciences, University of Siena, Siena, Italy; ^5^ Department of Biomedical Experimental and Clinical Sciences “Mario Serio”, University of Florence, Florence, Italy; ^6^ Medical Genetics Unit, Meyer University Hospital, Firenze, Italy; ^7^ Medical Genetics Unit, Department of Medical Sciences and Public Health, University of Cagliari, Cagliari, Italy; ^8^ Department of Otorhinolaryngology, Careggi University Hospital, Florence, Italy; ^9^ Infectious and Tropical Diseases Unit, Careggi University Hospital, Florence, Italy

**Keywords:** CD40LG gene, CD40LG mutation, Leishmania, leishmaniasis, CPT2 deficiency, CPT2 gene, hyper-IgM immunodeficiency syndrome, WES - whole-exome sequencing

## Abstract

X-linked hyper-IgM (XHIGM) syndrome is caused by mutations of the CD40LG gene, encoding the CD40L protein. The clinical presentation is characterized by early-onset infections, with profound hypogammaglobulinemia and often elevated IgM, susceptibility to opportunistic infections, such as *Pneumocystis jirovecii* pneumonia, biliary tract disease due to *Cryptosporidium parvum*, and malignancy. We report a 41-year-old male presenting with recurrent leishmaniasis, hypogammaglobulinemia, and myopathy. Whole-exome sequencing (WES) identified a missense variant in the CD40LG gene (c.107T>A, p.M36K), involving the transmembrane domain of the protein and a missense variant in the carnitine palmitoyl-transferase II (CPT2; c.593C>G; p.S198C) gene, leading to the diagnosis of hypomorphic XHIGM and CPT2 deficiency stress-induced myopathy. A review of all the previously reported cases of XHIGM with variants in the transmembrane domain showcased that these patients could present with atypical clinical features. Variants in the transmembrane domain of CD40LG act as hypomorphic generating a protein with a lower surface expression. Unlike large deletions or extracellular domain variants, they do not abolish the interaction with CD40, therefore preserving some biological activity.

## Introduction

CD40 ligand (CD40L) deficiency is a combined immunodeficiency (CID), characterized by a defective T–B lymphocyte cross talk and class-switch recombination (CSR) (1), also called X-linked hyper-IgM syndrome (XHIGM). It is caused by mutations of the *CD40LG* gene located in the long arm of the X chromosome (Xq26.3) (2). CD40L, also known as CD154, is a transmembrane protein transiently expressed mainly on activated CD4+ T cells but also in other cell types. It binds CD40, also known as TNFRSF5, expressed on B lymphocytes, dendritic cells, and monocytes/macrophages. Acting as a costimulatory signal, the CD40–CD40L interaction mediates CSR in B cells and leads in APCs to the upregulation of MHC-II, LFA-3, B7, and B7-1 promoting antigen presentation, priming, and cross-priming of T helper cells and cytotoxic T lymphocytes, and cytokine release including secretion of IL-1, IL-6, IL-8, IL-12, TNF-α, and MIP-1α (3, 4). CD40L is a 32-kD protein of 261 amino acids, containing an intracytoplasmic tail (IC), a short transmembrane (TM) domain, and an extracellular portion (EC) with a TNF-homology domain (5). The CD40LG gene contains 5 exons; the first exon encodes for the IC and TM domains. Most reported variants in XHIGM are stop-gain, frameshift deletions, splicing variants, or missense variants in the extracellular domain, abolishing protein expression and/or interaction with CD40. A few variants have been reported in the IC and TM domains (6). The classic presentation of XHIGM includes childhood severe opportunistic infections, neutropenia, and liver disease. Serum IgG, IgA, and IgE are usually markedly reduced, while IgM can be increased or within normal limits (1). With the widespread use of unbiased next-generation sequencing approaches, like whole-exome (WES) and whole-genome sequencing (WGS), the phenotypic spectrum of several diseases is expanding. Herein, we review atypical and late-onset presentations of XHIGM syndrome and their genotype–phenotype correlation and report a new case of an adult presenting with recurrent mucocutaneous leishmaniasis, hypogammaglobulinemia, and recurrent elevated creatinine phosphokinase.

## Methods

*Whole-exome sequencing (WES)* was employed to search for genetic variants. DNA was extracted from peripheral whole blood. Libraries were made using the SeqCap EZ Exome v3 (Roche NimbleGen, Pleasanton, CA, USA) capture kit and sequenced on an Illumina NextSeq 550 platform. Reads were aligned to the reference genome Grch37 (hg19) using the Burrows–Wheeler Aligner (BWA). Variant calling was done using the GATK-unified genotyper module. Variant annotation was done using ANNOVAR. A panel of genes associated with inborn errors of immunity was used to prioritize candidate variants. The panel included the “Primary Immunodeficiency” PanelApp (version 2.384) gene panel and the genes in the 2019 IUIS IEI classification.

*Lymphocyte populations* and subpopulations were measured from fresh EDTA blood samples using multicolor flow cytometry panels (**Supplementary Table 1**). Data were acquired on a FACSCanto II (BD Biosciences, Franklin Lakes, NJ, USA) and analyzed using FACSDiva software (BD Biosciences).

*CD40L expression* was measured on peripheral blood mononuclear cells (PBMC) separated by density gradient centrifugation (Ficoll). PBMCs were stimulated with phorbol 12-myristate 13-acetate (PMA) and ionomycin for 2 h, and membrane staining was done using PE-conjugated anti-CD40L (BD Biosciences), FITC-conjugated anti-CD4 (BD Biosciences), V450-conjugated anti-CD3 (BD Biosciences), and APC-conjugated anti-CD8 (BD Biosciences). Data were acquired on a BD LSR II flow cytometer (BD Biosciences) and analyzed with the FlowJo software (BD Biosciences).

*T-cell proliferation assay* was performed after fluorescent intracellular *labeling* with carboxyfluorescein succinimidyl ester (CFSE): briefly, PBMCs were stained with CFSE and after an overnight incubation at 37°C 5% CO_2_ were stimulated with or without phytohemagglutinin (PHA) 1% or anti-human CD3/CD28 monoclonal antibodies for 3 and 5 days. Data were acquired on a BD LSR II flow cytometer (BD Biosciences) and analyzed by the FACSDiva software (BD Biosciences). Relative proliferation was calculated for total CD3+ lymphocytes and separately for CD4+ and CD8+ T lymphocytes using the difference between the geometric mean of the stained non-proliferating cells and the geometric mean of the total stained population.

*Cytokine production* was evaluated by intracytoplasmic staining. Briefly, T cells were stimulated with PMA plus ionomycin for 6 h, the last three in the presence of brefeldin A. Cells were then fixed in formaldehyde and analyzed for intracellular cytokine production on a BD LSR II flow cytometer (BD Biosciences). Data were analyzed with the FlowJo software (BD Biosciences). Fluorochrome-conjugated monoclonal antibodies are listed in **Supplementary Table 1**.

ClinVar and HGMD databases were searched for pathogenic *CD40LG* variants. We used PubMed using the keywords “CD40LG deficiency,” “CD40L deficiency,” and “Hyper-IgM syndrome,” and the manuscripts referenced within those papers for the literature review.

*Muscle biopsy* specimens from a surgical biopsy of the *rectus femoralis* muscle were snap-frozen in isopentane chilled in liquid nitrogen. Cryostat sections were submitted to routine histological and histochemical stains, as well as to immunohistochemistry for MHC I, MHC II, and complement membrane attack complex (MAC).

## Results

**Figure 1:**
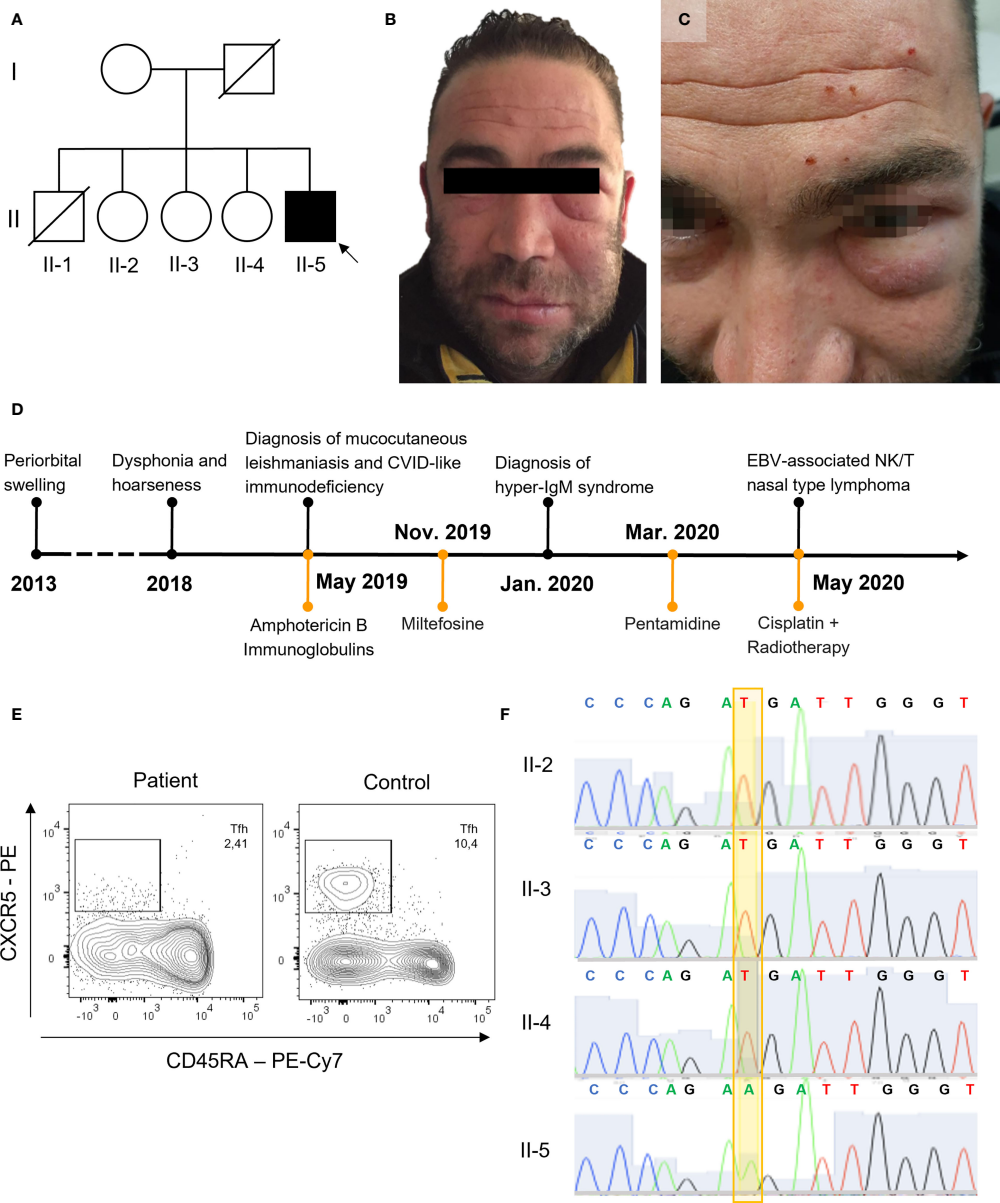
Clinical presentation and genetic analysis. **(A)** Family pedigree, black background used for CD40LG variant. **(B, C)** Periorbital swelling. **(D)** Timeline of clinical events and therapy. **(E)** Tfh in the index patient and a representative control (gated on CD3+CD4+ lymphocytes from whole-blood). **(F)** Sanger sequencing of *CD40LG* (NM_000074) c.107T>A p.Met36Lys.

*Case presentation.* A 41-year-old Caucasian male with laryngeal and facial mucocutaneous leishmaniasis in treatment with intravenous liposomal amphotericin B was admitted to our hospital because of elevated creatinine kinase (CK)—more than 100 upper limit of normal—in the absence of myalgia or muscle weakness. He was born to non-consanguineous parents. His father died in his 60s from gastric adenocarcinoma, while his mother was in good health. His brother died at 20 years of age from an unspecified lymphoproliferative disease. He had three sisters, and one of them suffered from chronic myalgias (**Figure 1A**). He worked as a construction worker. He lived in a small town in the province of Florence, Italy, and reported having previously traveled in Spain and France for vacation, while he denied having never traveled outside Europe. His past medical history was notable for visceral leishmaniasis at age 13, recurrent otitis and an episode of bronchopneumonia during childhood, and unexplained elevated CK on several occasions that were attributed to his occupation and to boxing that he played in his spare time. In 2013, at the age of 35, the patient developed periorbital swelling of the left side of the face, associated with mild erythema and skin thickening that was diagnosed as Morbihan’s disease. The swelling did not improve with retinoid treatment and transiently responded to low-dose steroid (**Figure 1B**). A few years later, in January 2019 at the age of 40, hoarseness and dysphonia appeared. Laryngoscopy found lesions involving the false right cord, the true vocal cords, and the anterior commissure. Histopathology and PCR of the laryngeal biopsies were positive for *Leishmania* spp. Retrospectively, the paraffin-included cutaneous samples from the left lower eyelid in 2015 were also found to be positive by PCR for *Leishmania* spp. Leishmania serology and PCR for leishmania on blood (immunofluorescence antibody test) were negative.

A diagnosis of mucocutaneous leishmaniasis was made. The patient was treated with intravenous liposomal amphotericin B (4 mg/kg/day for a total of 14 infusions), but after the first infusions he developed hyperCKemia. The pretreatment levels of CK were slightly elevated up to twice the upper limit normal (ULN). Upon admission, the CK levels rose to over ten times the ULN and were associated with an increase in serum creatinine. For this reason, in April 2019 he was admitted to our hospital for further evaluation and an immunology consult was requested in the suspicion of dermatomyositis. On examination, the patient presented in good general conditions. He had bilateral orbital swelling (**Figure 1C**). An electromyography and successively a muscle biopsy were performed to exclude an inflammatory myopathy, despite the long-standing history of elevated CK. Myositis-associated and specific autoantibodies were negative. Nonetheless, because of the *Leishmania* infection, the suspicion of an immunodeficiency was raised. A timeline of clinical events is reported in **Figure 1D**. Peripheral complete blood count was unremarkable, and he had normal T CD4+ and slightly elevated T CD8+ counts with a reduced CD4+/CD8+ ratio and reduced NK and B cells. Serum IgG levels were reduced, with borderline low IgA and normal to borderline elevated IgM. The B lymphocyte subpopulation showed a defect in the memory compartment, with markedly low switched-memory B cells (**Supplementary Table 2**). T-lymphocyte subpopulations were within the reference limits, except for reduced Tfh cells (**Figure 1E** and **Supplementary Table 2**). There was no history of liver and biliary tract disease or neutropenia. The patient was meeting criteria for CVID, even if he did not have a clear history of respiratory tract infections. However, because of the opportunistic persistent infection, further genetic studies were sought.

**Figure 2:**
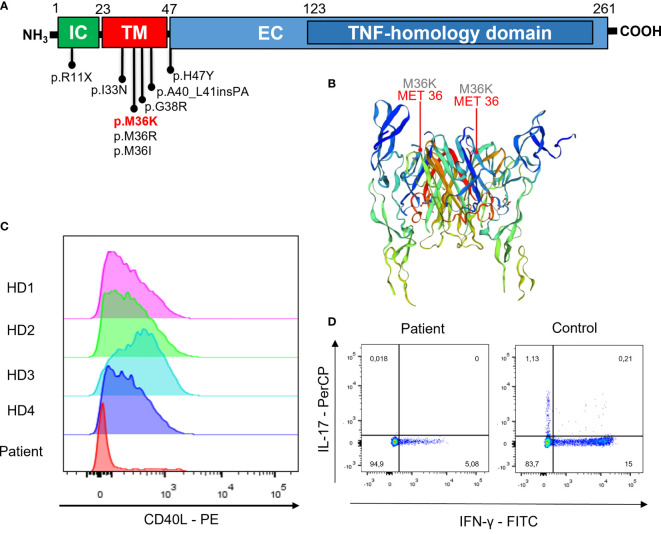
Gene model, CD40L expression, and cytokine production. **(A)** Localization of variants associated to atypical phenotypes and in the transmembrane domain. **(B)** Crystal structure of CD40L (P25942) from https://swissmodel.expasy.org/repository/uniprot/P25942?template=3qd6. **(C)** CD40L expression after polyclonal stimulation (histogram plots show cells gated on CD3+CD4+ T cells from PBMNCs) in the patient and in four different healthy donors (HD). **(D)** IFN-γ and IL-17 intracellular staining after polyclonal stimulation in the patient and a representative control (dot plots show cells gated on CD3+CD4+ lymphocytes from PBMNCs).

WES identified a missense variant in the *CD40LG* gene (c.107T>A, p.M36K). The variant was confirmed by Sanger sequencing (**Figure 1F**) and was found only in the proband. It was not possible to test his deceased brother, but the clinical history of early-onset lymphoproliferative disease was highly suggestive that he might have been affected as well. This variant is localized in the transmembrane domain of the CD40L protein (**Figures 2A, B**). It is absent in large-population databases (gnomAD, 1000k genomes) and is reported as “likely pathogenic” in ClinVar. The residue is evolutionarily conserved (GERP++ 4.51), and the substitution is computationally predicted to be deleterious (CADD 23.7). A missense variant in the same residue (p.M36R) was previously reported as pathogenic by Korthauer et al. (**Table 1**). Functional validation was performed by CD40L staining after polyclonal stimulation with PMA/ionomycin. The expression of CD40L on activated T CD4+ cells was confirmed to be significantly reduced (27% with reference value between 40% and 80%) (**Figure 2C**). Moreover, secretion of IFN-γ from stimulated T CD4+ was also reduced (5.1% with reference value 9.6%–44.8%) (**Figure 2D** and **Supplementary Figure S1**). T-cell proliferation to PHA and anti-CD3/CD28 was not reduced (**Supplementary Figure S2**).

**Table 1:** CD40LG transmembrane domain variants and selected variants that have been reported to carry a mild/atypical phenotype.

Domain	Coding DNA	Protein		Source	Phenotype
**IC**	c.31C>T	p.R11X	Arg > Ter	Kiani-Alikhan et al. (7)	Mild XHIGM
**TM**	c.98T>A	p.I33N	Ile > Asn	Thaventhiran et al. (8)	CVID
**TM**	c.107T>A	p.M36K	Met > Lys	ClinVar and this report	HIGM
**TM**	c.107T>G	p.M36R	Met > Arg	Korthauer et al. (9)	HIGM
**TM**	c.108G>A	p.M36I	Met > Ile	ClinVar	VUS
**TM**	c.112G>C	p.G38R	Gly > Arg	Katz et al. (10)	HIGM
**TM**	c.116_121dupCAGCAC	p.A40_L41insPA	Ins Pro-Ala	França et al. (11)	Mild XHIGM
**EC**	c.139C>T	p.H47Y	Hys > Tyr	Günaydin et al. (12)	Mild XHIGM
**EC**	c.761C>T	p.T254M	Thr > Met	Seyama et al. (13)	XHIGM

CVID, common variable immunodeficiency; HIGM, hyper-IgM syndrome; VUS, variant of unknown significance; IC, intracellular; TM, transmembrane; EC, extracellular.

In the meantime, the muscle biopsy excluded the inflammatory nature of the muscle disease; no immune-histological changes of membrane-associated proteins nor pathological lipid or glycogen storage were detected (**Supplementary Figures 3A–C**). In consideration of a possible metabolic myopathy with no surcharge, genes associated with hyperCKemia were investigated. A heterozygous missense variant in the carnitine palmitoyl-transferase II (*CPT2*; c.593C>G; p.S198C) was found (**Supplementary Figure 3D**). This variant is not reported in the general population and predicted to be deleterious by multiple bioinformatic tools. Heterozygous *CPT2* variants can cause adult-onset stress-induced myopathy (OMIM #255110). Among stressors, drugs, infections, exercise, cold, fasting, and trauma are included. The *CPT2* variant was also found in two of his sisters, and one of them had a history of elevated CK and myalgias (**Supplementary Figure 3E**).

The patient was started on immunoglobulin replacement therapy after finding the hypogammaglobulinemia. The leishmanial laryngeal involvement regressed within a few months after treatment with intravenous liposomal amphotericin B treatment (4 mg/kg/day, for a total of 15 infusions in a 6-month period). Due to the persistence of facial swelling, the patient was additionally treated with oral miltefosine 50 mg *tid* for 3 months and then intravenous pentamidine 4 mg/kg per day for 14 days and finally monthly maintenance therapy with intravenous pentamidine 4 mg/kg which is still ongoing.

During the follow-up (in May 2020), the facial swelling completely resolved; however, the patient developed a soft palate lesion that on biopsy was diagnosed as extra-nodal NK/T nasal-type EBV-associated lymphoma. A biopsy of the pharyngeal wall lesion indeed showed a lymphoid proliferation with cytoplasmic CD3+, CD2+, CD7-, CD56-, CD20-, CD5-, granzyme+, TIA-1+, Ki67 90%, EBER diffusely and intensively positive. A bone marrow biopsy was negative for lymphoproliferation and showed normal hematopoietic progenitors. He underwent chemotherapy with cisplatin and local radiotherapy achieving a complete remission. He is currently in evaluation for bone marrow transplantation and is continuing monthly intravenous pentamidine prophylaxis and immunoglobulin replacement therapy.

## Discussion

X-linked hyper-IgM syndrome is usually diagnosed in childhood, in patients presenting with severe infections, neutropenia, liver disease, and elevated IgM. Atypical forms of XHIGM can have an initial partial or milder presentation than typical forms, but later worsening of the clinical picture can occur as seen in this case report. Therefore, the recognition of XHIGM in adults can be challenging. Our patient had normal or only mildly elevated IgM and no history of neutropenia, liver disease, or severe respiratory infections. Without the typical features, the presentation can mimic common variable immunodeficiency (CVID) or combined immunodeficiency (CID). In hindsight, the reduction of circulating Tfh cells was among the only laboratory features pointing at the correct diagnosis. Tfh are generally elevated in CVID, and a reduction was typically described in patients with ICOS, BTK, and CD40L deficiency (14, 15). Notably, the patient also presented an inverted CD4/CD8 ratio with prominent T CD8+ senescence (CD57+), similarly to the case reported by Lougaris et al.; however, proliferative responses to mitogens (PHA and anti-CD3/CD28) were normal (16). Unbiased genetic approaches, such as WES and WGS, can help identify XHIGM in adults with CVID-like diseases.

Single-nucleotide missense variants in the transmembrane domain of CD40L generate a protein with a lower surface expression than the wild type; however, unlike large deletions or extracellular domain variants, they do not abolish the interaction with CD40. Hypomorphic variants in CD40LG can present with atypical clinical features, lacking the severe clinical presentation of classical XHIGM. This could explain the atypical and late-onset phenotype observed in our case. Most pathogenic variants reported in CD40LG are missense variants in the extracellular domain or null alleles (stop-gain, frameshift).

Few mutations in the transmembrane domain have been reported, and most of those insert a charged amino acid in the protein sequences disrupting the protein membrane expression (p.M36R, p.G38R) (**Table 1**). Among those, the reported phenotypes are often atypical or mimicking CVID. The p.I33N variant was found in a patient with recurrent bacterial infections, low IgG, IgA, and IgM, immune thrombocytopenia, autoimmune hepatitis, cutaneous vasculitis, increased TCRαβ DNT cells, and sensorineural hearing loss that was diagnosed as CVID (8). A small 6-nucleotide duplication leading to an in-frame insertion of proline and alanine in position 40 was recently reported to cause a mild hyper-IgM phenotype (c.116_121dupCAGCAC) (11). Another case of mild hyper-IgM was reported with p.H47Y which is the first amino acid of the extracellular domain (12). p.R11X introduces a stop codon in the first exon, encoding the intra-cytoplasmic portion of the protein. However, it was shown that this mutation allows re-initiation of translation at the downstream methionine in position 21, resulting in a shorter protein lacking the intracytoplasmic tail (7, 13). Patients carrying this variant present with reduced surface CD40L and have atypical and late-onset phenotypes. In several patients, the presentation was chronic anemia from Parvovirus B19 infection (13, 17) and other late-onset opportunistic infections like cerebral toxoplasmosis (18). The p.T254M variant is located near the end of the protein in the TNF-homology domain, and it disrupts the protein conformational folding (5). It was initially reported in patients with “milder” disease (13), but this definition was disputed by De Vries et al. (19) and Danielian et al. (20), as several patients displayed a severe clinical course, even so often atypical. Splicing donor site mutations leading to exon 2 skipping, such as c.309+2T>A, have also been associated with a milder phenotype. The produced shorter protein was shown to have a reduced binding affinity for CD40 (13). Skewed X-lyonization was described as a possible mechanism for atypical late-onset disease in female carriers of X-linked diseases. Although carriers of XHIGM are generally asymptomatic as a small population of cells expressing CD40L is sufficient to maintain normal humoral immunity (21), in extreme cases of X-lyonization a partial CD40L deficiency can appear mimicking CVID (22). Spontaneous somatic mutations resulting in revertant mosaicism have also been described as a cause for atypical phenotypes in patients with immunodeficiency; however, we are unaware of any such case described for CD40LG deficiency (23). The clinical spectrum of CD40L deficiency and the genotype–phenotype associations hint to the presence of a dose-dependent CD40L role in immune homeostasis. Notably, it was also described how the duplication of the *CD40LG* gene causes autoimmunity, as reported by Le Coz et al. (24).

Since the first description of visceral leishmaniasis in XHIGM syndrome by Martìn et al. in 1996, only a few cases have been reported so far in the literature (25–27). CD40L deficiency’s main feature is hypogammaglobulinemia, caused by the central role of the CD40–CD40L interaction in the class-switch recombination in B cells. However, CD40L also plays a critical role in Th1 differentiation by inducing the production of IL-12 by monocytes and dendritic cells (28, 29). Considering that a Th1 cell-mediated response is classically needed for *Leishmania* clearance (30), CD40L-deficient patients have an increased susceptibility to *Leishmania* species infections, as also seen in the animal experimental models. While in the murine model *Leishmania major* infection has been originally used to define the Th1/Th2 cell polarization paradigm, further studies in the experimental model and in humans have shown complex and dynamic immune responses (31–33). The importance of IFN-γ immunity in the control of leishmaniasis is also proven by previous case reports describing patients with defects in the IL-12/IFN-γ circuitry (34, 35). Among other inborn errors of immunity (IEI), leishmaniasis was also reported in chronic granulomatous disease (CGD), which is characterized by neutrophil dysfunction. Remarkably, a non-redundant role of the CD40L–CD40 interaction in neutrophil development and function mediated by IFN-γ was described (4).

This report also showcases the utility of WES data reanalysis and how it can help in revealing the unexpected co-occurrence of multiple genetic diseases. In this case, after finding a myopathic pattern on histopathology, WES was reanalyzed with a dedicated myopathy panel discovering a *CPT2* variant and leading to the diagnosis of carnitine palmitoyl-transferase II deficiency stress-induced myopathy (36).

A major clinical problem is that the translation of pediatric management guidelines to adult patients with atypical forms is not straightforward. Hematopoietic stem cell transplantation (HSCT) is the only curative treatment for CD40L deficiency, and its success depends on a prompt diagnosis. In a recent international collaborative study, the outcome improved if HSCT was performed before organ damage development, particularly considering sclerosing cholangitis and infections (37). However, when balancing the risks of HSCT with long-term supportive therapy, no differences in survival have been observed, although patients treated with HSCT have higher Karnofsky/Lansky performance status than those not receiving HSCT (38). Patients for whom HSCT may not represent an adequate treatment option, such as those with chronic treatment-resistant infections, are clinically very challenging and call for innovative treatment options.

The takeaway message of this report is the central role of WES as a diagnostic tool in IEI and the importance of opportunistic infections as red flags, independently from the age of the patient. The discovery of novel variants is constantly expanding the phenotypic spectrum of well-known diseases. In this report, we have shown how Leishmania infection led to the unveiling of a previously unrecognized CD40L deficiency in an adult patient. Reviews of previously published *CD40LG* mutations in the transmembrane domain hint to the presence of a genotype–phenotype correlation with atypical XHIGM forms. This could be explained by the reduced surface CD40L protein expression. WES reanalysis enabled the serendipitous discovery of carnitine palmitoyl-transferase II deficiency stress-induced myopathy in the same patient. Finally, treatment-refractory opportunistic infections, as in this case, should always alert to a serious immune dysfunction and warrant case discussion within a multidisciplinary group.

## Data Availability Statement

Genetic polymorphism data have been deposited in the European Variation Archive (EVA) at EMBL-EBI under accession number PRJEB52220 (https://wwwdev.ebi.ac.uk/ena/browser/view/PRJEB52220).

## Ethics Statement

The studies involving human participants were reviewed and approved by the Ethics Committee of the “Area Vasta Centro”, Florence, Italy (Ref CEAVC 13096). The patients/participants provided their written informed consent to participate in this study. Written informed consent was obtained from the individual(s) for the publication of any potentially identifiable images or data included in this article.

## Author Contributions

BP and LS drafted the manuscript. VM cared for the patient and collected the clinical data. MC, LM, and AM designed and performed the experiments and analyzed the data. FL and LZ cared for the patient, provided clinical data, conceptualized the paper, and critically reviewed the manuscript. LT, AP, and SG did the genetic testing. NV reviewed the manuscript and provided the muscle biopsy data. LC, FA, and PP conceptualized the paper and critically reviewed the manuscript. All authors contributed to the article and approved the submitted version.

## Funding

The CVIDome project was funded in Call for Research Projects 2019 by “Fondazione Cassa Risparmio di Firenze” and in the “BANDO RICERCA SALUTE 2018” by the Regione Toscana.

## Conflict of Interest

The authors declare that the research was conducted in the absence of any commercial or financial relationships that could be construed as a potential conflict of interest.

## Publisher’s Note

All claims expressed in this article are solely those of the authors and do not necessarily represent those of their affiliated organizations, or those of the publisher, the editors and the reviewers. Any product that may be evaluated in this article, or claim that may be made by its manufacturer, is not guaranteed or endorsed by the publisher.

## Supplementary Material

Supplementary Figure 1 IL-4 and IFN-γ intracellular staining following polyclonal stimulation in the patient and a representative control (dots plots show cells gated on CD3+CD4+ T cells from PBMNCs).

Supplementary Figure 2 T cell proliferation to mitogens **(A)** patient and a representative control, on CD4+ and CD8+ T cells from PBMCs stimulated with PHA 1:100 or anti-CD3/CD28 antibodies. **(B)** Relative proliferation expressed as the difference between the geometric mean of the non-proliferating stained cells and the geometric mean of the total stained cells.

Supplementary Figure 3 CPT2 deficiency stress-induced myopathy. **(A)** Muscle biopsy showed normal muscle fibers with no atrophy, necrosis or inflammation (H&E) with **(B)** normal distribution of MHC-I and HLA-ABC complex and **(C)** moderate acid phosphatase reactivity; **(D)** Sanger sequencing of *CPT2* (NM_000098): c.[593C>G];[=], p.[Ser198Cys];[=]; **(E)** Family pedigree gray background used for CPT2 variant.

Supplementary Table 1 List of all fluorochrome monoclonal antibodies used for flow cytometric analysis.

Supplementary Table 2 Clinical laboratory data.

## Abbreviations

XHIGM, X-linked hyper-IgM; WES, whole-exome sequencing; CPT2, carnitine palmitoyl-transferase II; CSR, class-switch recombination; WGS, whole-genome sequencing; BWA, Burrows–Wheeler Aligner; GATK, Genome Analysis Toolkit; PBMC, peripheral blood mononuclear cells; PMA, phorbol 12-myristate 13-acetate; BFA, brefeldin A; CK, creatinine kinase; PCR, polymerase chain reaction; CVID, common variable immunodeficiency; EBV, Epstein–Barr virus; CID, combined immunodeficiency; Tfh, T follicular helper lymphocytes; DNT, double-negative T cells.

## References

[1] Leven EA Maffucci P Ochs HD Scholl PR Buckley RH Fuleihan RL. Hyper IgM Syndrome: A Report From the USIDNET Registry. J Clin Immunol (2016) 36(5):490–501. doi: 10.1007/s10875-016-0291-4 27189378 PMC5039943

[2] Yazdani R Fekrvand S Shahkarami S Azizi G Moazzami B Abolhassani H. The Hyper IgM Syndromes: Epidemiology, Pathogenesis, Clinical Manifestations, Diagnosis and Management. Clin Immunol (2019) 198:19–30. doi: 10.1016/j.clim.2018.11.007 30439505

[3] Kracker S Di Virgilio M Schwartzentruber J Cuenin C Forveille M Deau M-C. An Inherited Immunoglobulin Class-Switch Recombination Deficiency Associated With a Defect in the INO80 Chromatin Remodeling Complex. J Allergy Clin Immunol (2015) 135:998–1007.e6. doi: 10.1016/j.jaci.2014.08.030 25312759 PMC4382329

[4] Cabral-Marques O França TT Al-Sbiei A Schimke LF Khan TA Feriotti C. CD40 Ligand Deficiency Causes Functional Defects of Peripheral Neutrophils That are Improved by Exogenous IFN-γ. J Allergy Clin Immunol (2018) 142(5):1571–88.e9. doi: 10.1016/j.jaci.2018.02.026 PMC6123297 29518426

[5] Thusberg J Vihinen M. The Structural Basis of Hyper IgM Deficiency – CD40L Mutations. Protein Engineer Design Selection (2007) 20:133–41. doi: 10.1093/protein/gzm004 17307885

[6] Garber E Su L Ehrenfels B Karpusas M Hsu Y-M. CD154 Variants Associated With Hyper-IgM Syndrome Can Form Oligomers and Trigger CD40-Mediated Signals. J Biol Chem (1999) 274:33545–50. doi: 10.1074/jbc.274.47.33545 10559240

[7] Kiani-Alikhan S Yong PFK Gilmour KC Grosse-Kreul D Davies EG Ibrahim MAA. Phenotypic Heterogeneity in a Family With a CD40 Ligand Intracellular Domain Mutation. J Clin Immunol (2012) 32:70–7. doi: 10.1007/s10875-011-9607-6 22009004

[8] Thaventhiran JED Lango Allen H Burren OS Rae W Greene D Staples E. Whole-Genome Sequencing of a Sporadic Primary Immunodeficiency Cohort. Nature (2020) 583:90–5. doi: 10.1038/s41586-020-2265-1 PMC7334047 32499645

[9] Korthäuer U Graf D Mages HW Brière F Padayachee M Malcolm S. Defective Expression of T-Cell CD40 Ligand Causes X-Linked Immunodeficiency With Hyper-IgM. Nature (1993) 361(6412):539–41. doi: 10.1038/361539a0 7679206

[10] Katz F Hinshelwood S Rutland P Jones A Kinnon C Morgan G. Mutation Analysis in CD40 Ligand Deficiency Leading to X-Linked Hypogammaglobulinemia With Hyper IgM Syndrome. Hum Mutat (1996) 8(3):223–8. doi: 10.1002/(SICI)1098-1004(1996)8:3<223::AID-HUMU5>3.0.CO;2-A 8889581

[11] França TT Leite LFB Maximo TA Lambert CG Zurro NB Forte WCN. Condino-Neto A. A Novel De Novo Mutation in the CD40 Ligand Gene in a Patient With a Mild X-Linked Hyper-IgM Phenotype Initially Diagnosed as CVID: New Aspects of Old Diseases. Front Pediatr (2018) 6:130. doi: 10.3389/fped.2018.00130 29780795 PMC5945832

[12] Günaydin NC Chou J Karaca NE Aksu G Massaad MJ Azarsiz E. A Novel Disease-Causing CD40L Mutation Reduces Expression of CD40 Ligand, But Preserves CD40 Binding Capacity. Clin Immunol (2014) 153:288–91. doi: 10.1016/j.clim.2014.05.001 24845792

[13] Seyama K Kobayashi R Hasle H Apter AJ Rutledge JC Rosen D. Parvovirus B19-Induced Anemia as the Presenting Manifestation of X-Linked Hyper-IgM Syndrome. J Infect Dis (1998) 178:318–24. doi: 10.1086/515633 9697710

[14] Coraglia A Galassi N Fernández Romero DS Juri MC Felippo M Malbrán A. Common Variable Immunodeficiency and Circulating TFH. J Immunol Res (2016) 2016:4951587. doi: 10.1155/2016/4951587 27069935 PMC4812460

[15] Ma CS Wong N Rao G Avery DT Torpy J Hambridge T. Monogenic Mutations Differentially Affect the Quantity and Quality of T Follicular Helper Cells in Patients With Human Primary Immunodeficiencies. J Allergy Clin Immunol (2015) 136:993–1006.e1. doi: 10.1016/j.jaci.2015.05.036 26162572 PMC5042203

[16] Lougaris V Lanzi G Baronio M Gazzurelli L Vairo D Lorenzini T. Progressive Severe B Cell and NK Cell Deficiency With T Cell Senescence in Adult CD40L Deficiency. Clin Immunol (2018) 190:11–4. doi: 10.1016/j.clim.2018.02.008 29476811

[17] Blaeser F Kelly M Siegrist K Storch GA Buller RS Whitlock J. Critical Function of the CD40 Pathway in Parvovirus B19 Infection Revealed by a Hypomorphic CD40 Ligand Mutation. Clin Immunol (2005) 117:231–7. doi: 10.1016/j.clim.2005.08.005 16169277

[18] Yong PFK Post FA Gilmour KC Grosse-Kreul D King A Easterbrook P. Cerebral Toxoplasmosis in a Middle-Aged Man as First Presentation of Primary Immunodeficiency Due to a Hypomorphic Mutation in the CD40 Ligand Gene. J Clin Pathol (2008) 61:1220–2. doi: 10.1136/jcp.2008.058362 18955577

[19] de Vries E Noordzij JG Davies EG Hartwig N Breuning MH van Dongen JJ. The 78c –> T (T254M) XHIM Mutation: Lack of a Tight Phenotype-Genotype Relationship. Blood (1999) 94:1488–90. doi: 10.1182/blood.V94.4.1488 10484640

[20] Danielian S Oleastro M Eva Rivas M Cantisano C Zelazko M. Clinical Follow-Up of 11 Argentinian CD40L-Deficient Patients With 7 Unique Mutations Including the So-Called “Milder” Mutants. J Clin Immunol (2007) 27:455–9. doi: 10.1007/s10875-007-9089-8 17351759

[21] Hollenbaugh D Wu LH Ochs HD Nonoyama S Grosmaire LS Ledbetter JA. The Random Inactivation of the X Chromosome Carrying the Defective Gene Responsible for X-Linked Hyper IgM Syndrome (X-HIM) in Female Carriers of HIGM1. J Clin Invest (1994) 94:616–22. doi: 10.1172/JCI117377 PMC296138 7518839

[22] de Saint Basile G Tabone MD Durandy A Phan F Fischer A Le Deist F. CD40 Ligand Expression Deficiency in a Female Carrier of the X-Linked Hyper-IgM Syndrome as a Result of X Chromosome Lyonization. Eur J Immunol (1999) 29:367–73. doi: 10.1002/(SICI)1521-4141(199901)29:01<367::AID-IMMU367>3.0.CO;2-4 9933119

[23] Wada T Candotti F. Somatic Mosaicism in Primary Immune Deficiencies. Curr Opin Allergy Clin Immunol (2008) 8:510–4. doi: 10.1097/ACI.0b013e328314b651 18978464

[24] Le Coz C Trofa M Syrett CM Martin A Jyonouchi H Jyonouchi S. CD40LG Duplication-Associated Autoimmune Disease Is Silenced by Nonrandom X-Chromosome Inactivation. J Allergy Clin Immunol (2018) 141:2308–311.e7. doi: 10.1016/j.jaci.2018.02.010 29499223 PMC5994181

[25] Martin JC Sancho T Sierra FJ Puig JG Lavilla P Jimenez C. Visceral Leishmaniasis in a Patient With Hyper-IgM Hypogammaglobulinemia. Clin Infect Dis (1996) 23:1188–9. doi: 10.1093/clinids/23.5.1188 8922833

[26] Gonzalez-Granado LI Dominguez-Pinilla N Gallego-Bustos F Ruiz-Contreras J Allende LM. Visceral Leishmaniasis May Unmask X-Linked Hyper-IgM Syndrome. J Clin Immunol (2016) 36:363–5. doi: 10.1007/s10875-016-0270-9 26984850

[27] Drabe CH Marvig RL Borgwardt L Lundgren JD Maquart HVH Katzenstein TL. Case Report: Hyper IgM Syndrome Identified by Whole Genome Sequencing in a Young Syrian Man Presenting With Atypical, Severe and Recurrent Mucosal Leishmaniasis. Front Immunol (2020) 11:567856. doi: 10.3389/fimmu.2020.567856 33013931 PMC7516301

[28] Kato T Hakamada R Yamane H Nariuchi H. Induction of IL-12 P40 Messenger RNA Expression and IL-12 Production of Macrophages *via* CD40-CD40 Ligand Interaction. J Immunol (1996) 156:3932–8. 8621933

[29] Kelsall BL Stüber E Neurath M Strober W. Interleukin-12 Production by Dendritic Cells. The Role of CD40-CD40L Interactions in Th1 T-Cell Responses. Ann N Y Acad Sci (1996) 795:116–26. doi: 10.1111/j.1749-6632.1996.tb52660.x 8958922

[30] Kaye P Scott P. Leishmaniasis: Complexity at the Host-Pathogen Interface. Nat Rev Microbiol (2011) 11;9(8):604–15. doi: 10.1038/nrmicro2608 21747391

[31] Lohoff M Gessner A Bogdan C Röllinghoff M. The Th1/Th2 Paradigm and Experimental Murine Leishmaniasis. Int Arch Allergy Immunol (1998) 115(3):191–202. doi: 10.1159/000023900 9531160

[32] Rossi M Fasel N. How to Master the Host Immune System? Leishmania Parasites Have the Solutions! Int Immunol Mar (2018) 30(3):103–11. doi: 10.1093/intimm/dxx075 PMC5892169 29294040

[33] Zijlstra EE. PKDL and Other Dermal Lesions in HIV Co-Infected Patients With Leishmaniasis: Review of Clinical Presentation in Relation to Immune Responses. PloS Negl Trop Dis (2014) 8(11):e3258. doi: 10.1371/journal.pntd.0003258 25412435 PMC4238984

[34] Khalid MB Lemos SG Myint-Hpu K Draper D Stoddard J Niemela JE. Ifnγr1 Deficiency Presenting With Visceral Leishmaniasis and Mycobacterium Avium Infections Mimicking HLH. Pediatr Allergy Immunol (2021) 33(1):e13653. doi: 10.1111/pai.13653 34407251 PMC9304970

[35] Parvaneh N Barlogis V Alborzi A Deswarte C Boisson-Dupuis S Migaud M. Visceral Leishmaniasis in Two Patients With IL-12p40 and IL-12rβ1 Deficiencies. Pediatr Blood Cancer (2017) 64(6). doi: 10.1002/pbc.26362 27873456

[36] Joshi PR Deschauer M Zierz S. Carnitine Palmitoyltransferase II (CPT II) Deficiency: Genotype–Phenotype Analysis of 50 Patients. J Neurol Sci (2014) 338:107–11. doi: 10.1016/j.jns.2013.12.026 24398345

[37] Ferrua F Galimberti S Courteille V Slatter MA Booth C Moshous D. Hematopoietic Stem Cell Transplantation for CD40 Ligand Deficiency: Results From an EBMT/ESID-IEWP-SCETIDE-PIDTC Study. J Allergy Clin Immunol (2019) 143:2238–53. doi: 10.1016/j.jaci.2018.12.1010 30660643

[38] de la Morena MT Leonard D Torgerson TR Cabral-Marques O Slatter M Aghamohammadi A. Long-Term Outcomes of 176 Patients With X-Linked Hyper-IgM Syndrome Treated With or Without Hematopoietic Cell Transplantation. J Allergy Clin Immunol (2017) 139:1282–92. doi: 10.1016/j.jaci.2016.07.039 PMC5374029 27697500

