# Supplementary figures and images for: Variants Disrupting CD40L Transmembrane Domain and Atypical X-Linked Hyper-IgM Syndrome: A Case Report With Leishmaniasis and Review of the Literature
Source: Front Immunol. 2022 Apr 28;13:840767. doi: 10.3389/fimmu.2022.840767 (PMC9096836; doi:10.3389/fimmu.2022.840767)

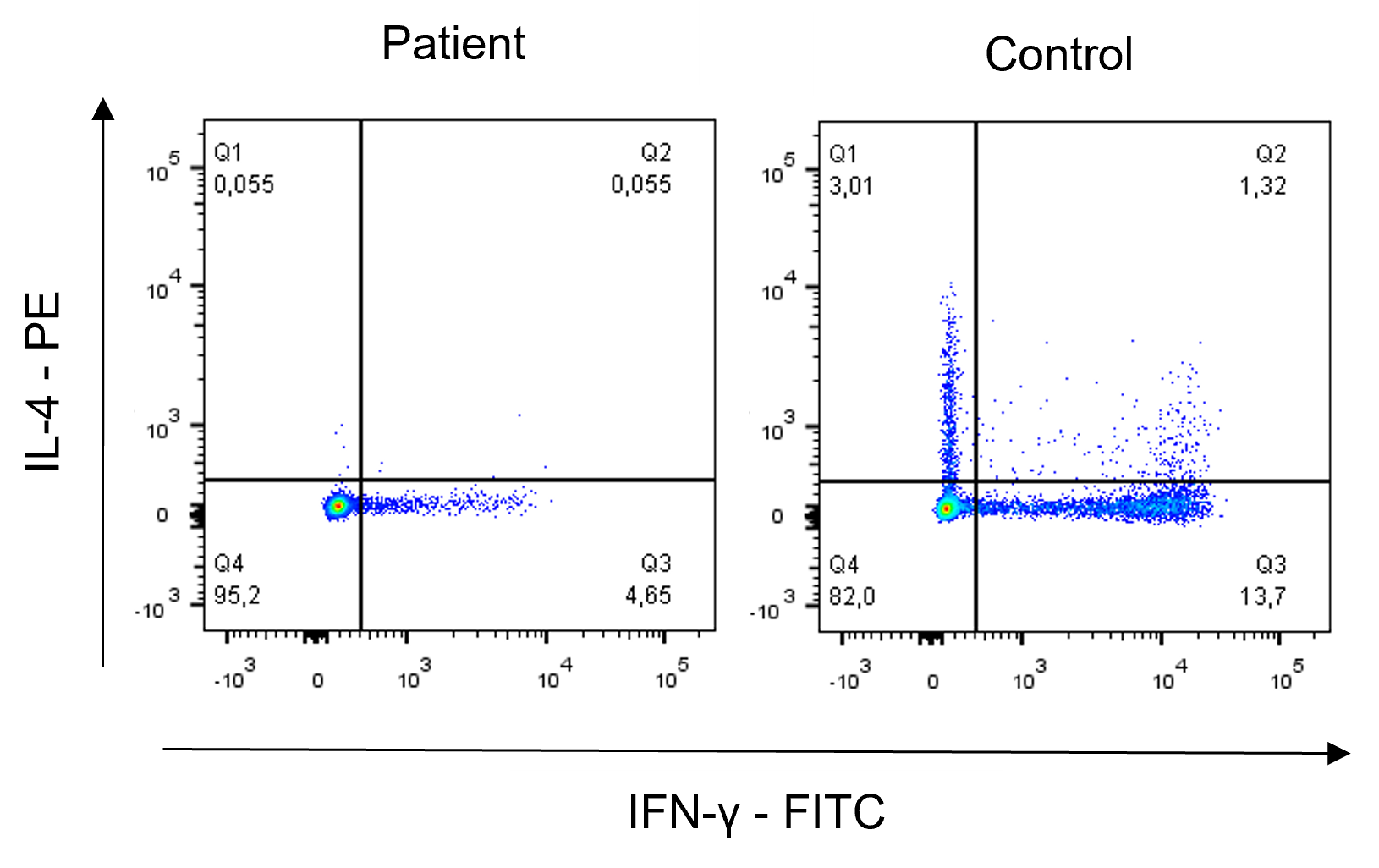

Supplement: Supplementary Figure 1 — IL-4 and IFN-γ intracellular staining following polyclonal stimulation in the patient and a representative control (dots plots show cells gated on CD3+CD4+ T cells from PBMNCs). [file Image_1.tif]

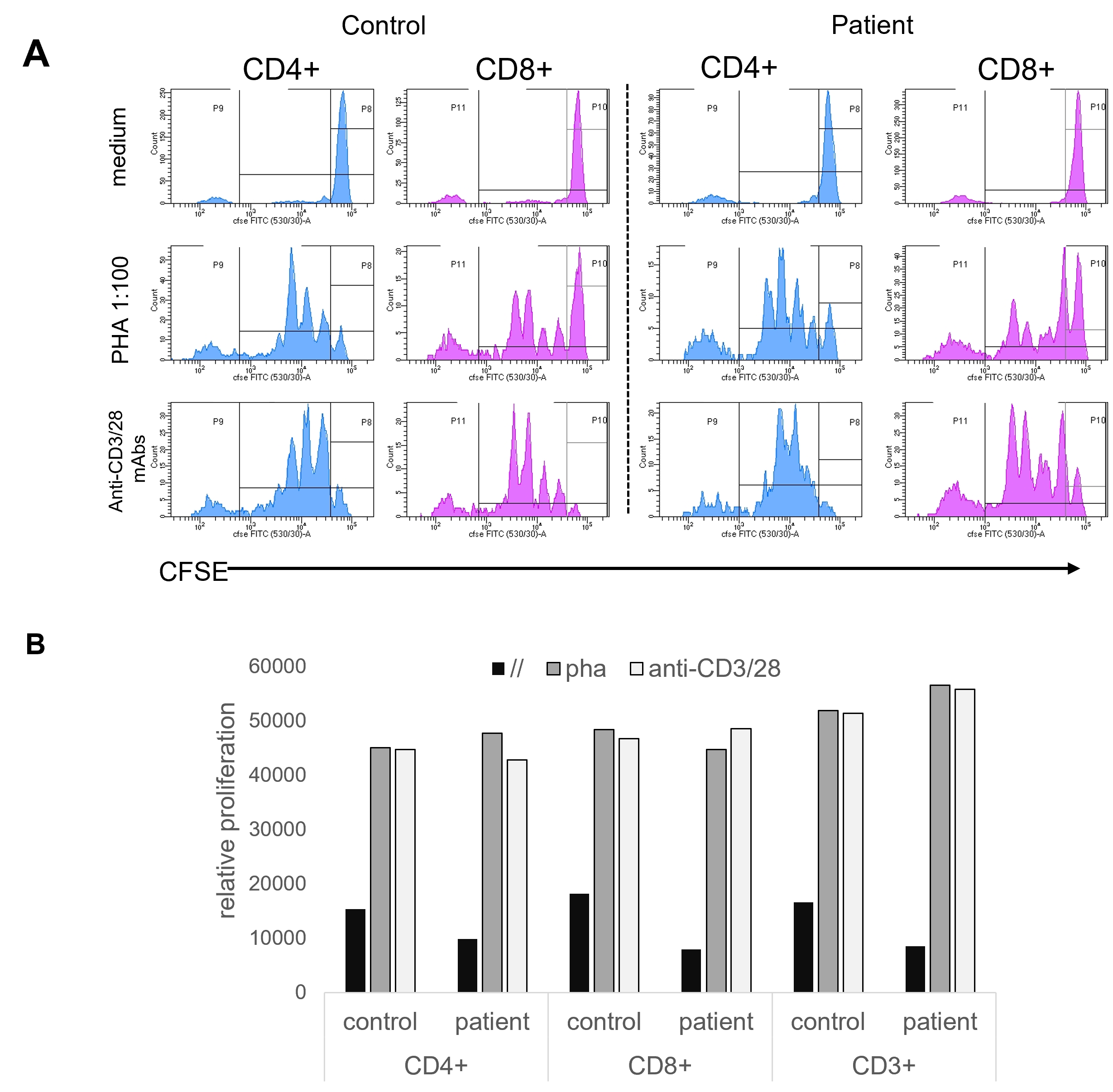

Supplement: Supplementary Figure 2 — T cell proliferation to mitogens (A) patient and a representative control, on CD4+ and CD8+ T cells from PBMCs stimulated with PHA 1:100 or anti-CD3/CD28 antibodies. (B) Relative proliferation expressed as the difference between the geometric mean of the non-proliferating stained cells and the geometric mean of the total stained cells. [file Image_2.tif]

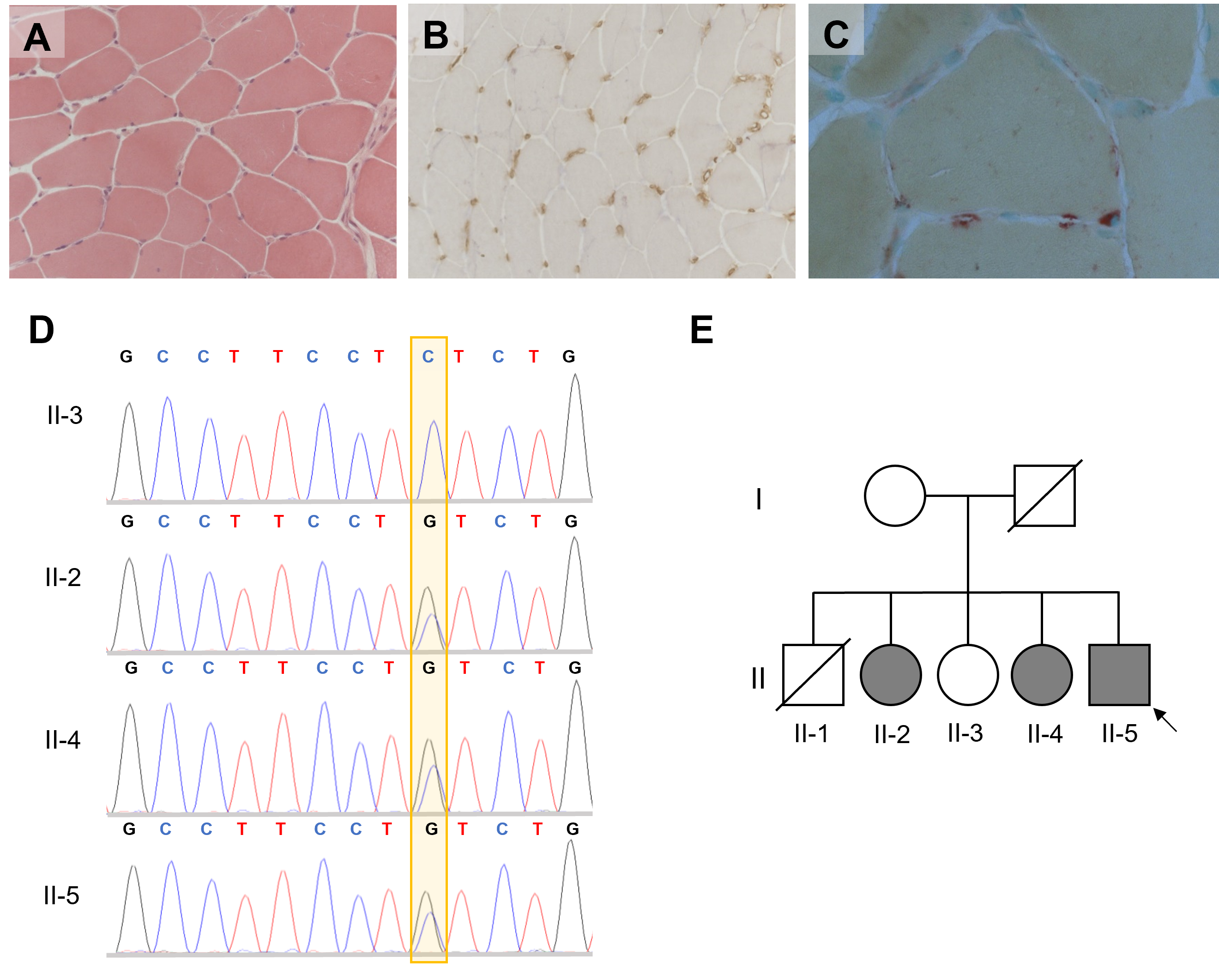

Supplement: Supplementary Figure 3 — CPT2 deficiency stress-induced myopathy. (A) Muscle biopsy showed normal muscle fibers with no atrophy, necrosis or inflammation (H&E) with (B) normal distribution of MHC-I and HLA-ABC complex and (C) moderate acid phosphatase reactivity; (D) Sanger sequencing of CPT2 (NM_000098): c.[593C>G];[=], p.[Ser198Cys];[=]; (E) Family pedigree gray background used for CPT2 variant. [file Image_3.tif]
