# Supplementary material for: Variants Disrupting CD40L Transmembrane Domain and Atypical X-Linked Hyper-IgM Syndrome: A Case Report With Leishmaniasis and Review of the Literature
Source: Front Immunol. 2022 Apr 28;13:840767. doi: 10.3389/fimmu.2022.840767 (PMC9096836; doi:10.3389/fimmu.2022.840767)
Supplement: Supplementary Table 1 — List of all fluorochrome monoclonal antibodies used for flow cytometric analysis. [file Table_1.docx]

**Supplementary Table S1. List of all fluorochrome monoclonal antibodies used for flow cytometric analysis.**

| **Antibodies** | **Fluorochromes** | **Producer** | **Clone** |
| --- | --- | --- | --- |
| IgM | FITC | BD PHARMINGEN | Mouse anti-IgM |
| IgD | PE | BD PHARMINGEN | Mouse anti-IgD |
| CD19 | PERCP | BD | SJ25C1 |
| CD38 | PECY7 | BD | HB7 |
| CD27 | APC | BD | L128 |
| CD20 | APCH7 | BD | L27 |
| CD21 | V450 | BD | BLY4 |
| CD45 | V500 | BD | 2D1 |
| TCRαβ | FITC | BD | WT31 |
| TCRγδ | PE | BD | 11F2 |
| CD3 | PERCP | BD | SK7 |
| CD25 | PECY7 | BD | 2A3 |
| CD8 | APC | BD | SK1 |
| CD4 | APCH7 | BD | SK3 |
| CD127 | V450 | BD HORIZON | HIL7RM21 |
| CD57 | FITC | BD | HNK1 |
| CD8 | PE | BD | SK1 |
| CD45RA | PECY7 | BD | L48 |
| CD31 | APC | BD PHARMINGEN | WM56 |
| CCR7 | V450 | BD HORIZON | 150503 |
| CXCR3 | FITC | R&D SYSTEM | 49801 |
| CXCR5 | PE | R&D SYSTEM | 51505 |
| CCR6 | APC | BD PHARMINGEN | 11AG |
