# Supplementary material for: Variants Disrupting CD40L Transmembrane Domain and Atypical X-Linked Hyper-IgM Syndrome: A Case Report With Leishmaniasis and Review of the Literature
Source: Front Immunol. 2022 Apr 28;13:840767. doi: 10.3389/fimmu.2022.840767 (PMC9096836; doi:10.3389/fimmu.2022.840767)
Supplement: Supplementary Table 2 — Clinical laboratory data. [file Table_2.docx]

**Supplementary Table S2. Clinical laboratory data.**

| **Variable** | **Reference Range, Adults** | **Patient,**  during hospital admission  (April 2019) |
| --- | --- | --- |
| **Blood** | | |
| Hematocrit (%) | 36–46 | 36.7 |
| Hemoglobin (g/dl) | 12–16 | 12.3 |
| Red-cell count (per μl) | 4200000–5400000 | 4420000 |
| Mean corpuscular volume (fl) | 81–94 | 83 |
| Red blood cell distribution width (%) | 11.8–14.8 | 15.5 |
| White‐cell count (per μl) | 4000–10000 | 5630 |
| Differential count (per μl) | | |
| Neutrophils | 1500–7500 | 2650 |
| Lymphocytes | 1000–4800 | 1910 |
| Monocytes | 300–1200 | 890 |
| Eosinophils | 0–700 | 170 |
| Basophils | 0–200 | 10 |
| Differential count (%) | | |
| Neutrophils | 37–75 | 47.1 |
| Lymphocytes | 12–50 | 33.9 |
| Monocytes | 3–12 | 15.8 |
| Eosinophils | 0–7 | 3 |
| Basophils | 0–2 | 0.2 |
| Platelet count (per μl) | 140000–440000 | 183000 |
| Prothrombin time (sec) | 9.5–12 | 13.7 |
| Prothrombin time (%) | 70-130 | 74 |
| Prothrombin‐time international normalized ratio | 0.8–1.2 | 1.21 |
| Activated-partial thromboplastin time (sec) | 22–38 | 36.6 |
| Activated-partial thromboplastin time (ratio) | 0.73–1.27 | 1.24 |
| Glucose (mg/dl) | 65-110 | 87 |
| Sodium (mEq/liter) | 135–145 | 142 |
| Potassium (mEq/liter) | 3.5–5.1 | 4.3 |
| Chloride (mEq/liter) | 95–110 | 105 |
| Calcium (mg/dl) | 8.6–10.2 | 9 |
| Magnesium (mg/dl) | 1.7–2.5 | 1.8 |
| Creatinine (mg/dl) | 0.5–1.10 | 1.44 |
| Blood urea nitrogen (g/liter) | 0.25-0.64 | 0.66 |
| Creatinine kinase (U/liter) | 35-232 | 3340 |
| C‐reactive protein (mg/liter) | <9 | <9 |
| Fibrinogen (mg/dl) | 200–400 | 203 |
| Alanine aminotransferase (U/liter) | 30-65 | 82 |
| Aspartate aminotransferase (U/liter) | 15-37 | 125 |
| Gamma-glutamyl transpeptidase (U/liter) | 5–85 | 41 |
| Total bilirubin (mg/dL) | 0.2–1 | 0.4 |
| Lactate dehydrogenase (U/liter) | 135–214 | 590 |
| Protein | | |
| Total (g/dl) | 6–8.2 | 6.7 |
| Albumin (%) | 55.8–66.1 | 63.7 |
| α1-globulins (%) | 2.9–4.9 | 5.6 |
| α2-globulins (%) | 7.1–11.8 | 12.3 |
| β1-globulins (%) | 4.7–7.2 | 5.5 |
| β2-globulins (%) | 3.2–6.5 | 4.2 |
| γ-globulins (%) | 11.1–18.8 | 8.7 |
| IgG (mg/dl) | 700–1600 | 375 |
| IgA (mg/dl) | 70-400 | 61 |
| IgM (mg/dl) | 40-230 | 227 |
| IgE (KU/L) | 0-85 | 33.2 |
| Antinuclear antibodies (Hep-2) IIF | <1:160 | 1:160 Speckled |
| ENA CTD-screen (ratio) | <0.7 | 0.1 |
| Myositis specific autoantibodies panel |  | Negative |
| (Mi-2α, Mi-2β, TIF1γ, MDA5, NXP2, SAE1, Ku, PM-Scl100, PM-Scl75, Jo-1, SRP, PL-7, PL-12, EJ, OJ, Ro-52, cN-1A) |  |  |
| Total lymphocytes (per μl) | 500–5000 | 1910 |
| CD3+ (per μl) | 570–2800 | 1740 |
| CD3+CD4+ (per μl) | 600–2000 | 690 |
| CD3+CD8+ (per μl) | 110–800 | 960 |
| CD3+HLA-DR+ (per μl) | 0–590 | 70 |
| CD4+/CD8+ (per μl) | 1–3.5 | 0.7 |
| CD19+ (per μl) | 100–500 | 50 |
| CD3-CD16+CD56+ (per μl) | 200–400 | 120 |
| CD19+ (%) | 6.4–22.6 | 2.5 |
| Naive (IgD+CD27-) (%) | 42.6–82.3 | 74.7 |
| Memory non-switched (IgD+CD27+) (%) | 7.4–32.5 | 7.1 |
| Memory switched (IgD-CD27+) (%) | 6.5–29.1 | 1.6 |
| Transitional (IgM++CD38++) (%) | 0.6–3.4 | 10.6 |
| CD21low (%) | 0.9–7.6 | 3.7 |
| Plasmablasts (CD27++CD38++) (%) | 0.4–3.6 | 0 |
| CD3+ TCRα/β+ (%) | 36–98 | 93.7 |
| CD3+ TCRγ/δ+ (%) | 0.83–11 | 6.2 |
| CD3+CD4+ (%) | 28–64 | 36.1 |
| CD4+ naive (CD45RA+CCR7+) (%) | 16–100 | 61.5 |
| CD4+ TCM (CD45RA-CCR7+) (%) | 18–95 | 23.8 |
| CD4+ TEM (CD45RA-CCR7-) (%) | 1–23 | 13.8 |
| CD4+ TEMRA (CD45RA+CCR7-) (%) | 0–6.8 | 0.9 |
| RTE (CD4+CD31+) (%) | 7–100 | 28.9 |
| Treg (CD25++CD127low) (%) | 4–17 | 4 |
| Tfh (CD45RA-CXCR5+) (%) | 5–15.6 | 2.41 |
| CD3+CD8+ (%) | 12–40 | 50.5 |
| CD8+ naive (CD45RA+CCR7+) (%) | 6–100 | 50.1 |
| CD8+ TCM (CD45RA-CCR7+) (%) | 1–20 | 1.4 |
| CD8+ TEM (CD45RA-CCR7-) (%) | 14–98 | 8 |
| CD8+ TEMRA (CD45RA+CCR7-) (%) | 7–53 | 40.5 |
| CD8+CD57+ (%) |  | 30.6 |
| **Urine** |  |  |
| Color | Yellow | Yellow |
| Clarity | Clear | Clear |
| pH | 5.0–6.5 | 6.5 |
| Specific gravity | 1.015–1.025 | 1.011 |
| Protein | Negative | Negative |
| Glucose | Negative | Negative |
| Ketones | Negative | Negative |
| Bilirubin | Negative | Negative |
| Nitrite | Negative | Negative |
| Leukocyte esterase | Negative | Negative |
| Red cells (per μl) | <20 | 0 |
| Leukocytes (per μl) | <18 | 0 |

RTE: recent thymic emigrants; TCM: T central memory cells; Tfh: T follicular helper; TEM: T effector memory cells; TEMRA: T effector memory RA+ cells.
